# Telomere Maintenance Genes are associated with Type 2 Diabetes Susceptibility in Northwest Indian Population Group

**DOI:** 10.1038/s41598-020-63510-w

**Published:** 2020-04-15

**Authors:** Itty Sethi, Varun Sharma, Indu Sharma, Gurvinder Singh, Gh. Rasool Bhat, A. J. S Bhanwer, Swarkar Sharma, Ekta Rai

**Affiliations:** 1 Human Genetics Research Group, School of Biotechnology, Shri Mata Vaishno Devi University, Katra, J&K 182320 India; 2 Department of Human Genetics, Guru Nanak Dev University, Amritsar, 143005 Punjab India

**Keywords:** Genetic association study, Genetic predisposition to disease

## Abstract

Telomere length attrition has been implicated in various complex disorders including Type 2 Diabetes (T2D). However, very few candidate gene association studies have been carried out worldwide targeting telomere maintenance genes. In the present study, variants in various critical telomere maintenance pathway genes for T2D susceptibility in Northwest Indian population were explored. With case-control candidate gene association study design, twelve variants from seven telomere maintenance genes were evaluated. Amongst these five variants, rs9419958 (*OBFC1*), rs4783704 (*TERF2*), rs16847897 (*TERC/LRRC31*), rs10936599 (*TERC/MYNN*), and rs74019828 (*CSNK2A2*) showed significant association with T2D (at *p*-value ≤ 0.003, threshold set after Bonferroni correction) in the studied population. *In silico* analyses of these variants indicated interesting functional roles that warrant experimental validations. Findings showed that variants in telomere maintenance genes are associated with pathogenesis of T2D in Northwest Indian population. We anticipate further, such candidate gene association studies in other Indian populations and worldwide would contribute in understanding the missing heritability of T2D.

## Introduction

Type 2 Diabetes (T2D) is a common progressive metabolic complex disorder, affecting 77 million individuals in India only^1^. Substantial evidences suggest that genetic factors strongly influence the risk of T2D^2–5^ and efforts are made worldwide in understanding these factors. An early endeavour to identify the genetic variants and loci was centred on candidate genes, often associated with insulin pathways^6^. Recent large scale Genome wide association studies (GWAS) over the past decade, have indicated ~144 genetic variants associated with T2D worldwide^7^. GWAS captured majority of the common alleles but yet all these together explain only ~20% heritability of the disease^3^. An extensive effort is warranted for the identification of other variants and genetic loci that are contributing in the disease etiology^3,8,9^. We believe answer to the major missing heritability components of the T2D lies in genetic heterogeneity and is dependent on diverse populations yet to be evaluated extensively. Further, it is important to overlap pathway based candidate gene study approach with GWAS to bring out signals that might have been masked due to stringent criteria in GWAS.

Telomere attrition pathway has been associated with many complex disorders including T2D^10,11^. Various functional studies have implicated telomere attrition in insulin resistance, impaired glucose tolerance, obesity, inflammation^12^ as well as it has been found as an independent risk factor for T2D^13,14^. In fact, T2D and telomere attrition share common environmental risk factors including stress, less physical activity, and sedentary life style^15^. The telomere length is regulated and maintained by protein complexes (reviewed in Sethi *et al*., 2016)^15^. Few studies have indicated that the genes involved in the regulation of telomere length might also have implication in the telomere length attrition in T2D individuals^16–20^. However, such studies are limited in number. This makes the study of genes involved in telomere attrition important candidates for evaluation of their role in T2D susceptibility. In addition, India is a pivot of ethnicities and a foremost contributor to the world population with ample diversity yet least explored of all region especially lacking GWAS data for most of these population groups. Thus, exploring the association of telomere maintenance genes in Indian population for T2D susceptibility is anticipated to contribute extensively in knowledge of T2D susceptibility, especially with respect to telomere biology.

With this background, the first case-control association study was performed to explore the role of telomere maintenance genes in the development of T2D in the North-west Indian population. Keeping account of potential functional implication of genes involved in telomere maintenance and regulation (reviewed in Sethi *et al*.)^15^ variants were selected in some of the genes from shelterin, CST (*CTC1, STN1* and *TEN1)* and telomerase complex. The selected genes and their variant were Telomerase RNA component *(TERC)* – rs16847897, rs10936599, rs10936601, Telomerase Reverse Transcriptase (*TERT)* – rs2736100, Casein Kinase 2 subunit alpha 2 *(CSNK2A2)* – rs74019828, Telomeric Repeat Binding Factor 2 *(TERF2)* – rs4783704, Telomerase Associated Protein 1 *(TEP1)* – rs3093872, rs4982038, rs3093921, Telomeric Repeat Binding Factor 1 *(TERF1)* – rs2010441, rs6982126 and Oligonucleotide/Oligosaccharide-Binding Fold-Containing Protein 1 *(OBFC1)* – rs9419958 (Supplementary Table S1).

## Result and Discussion

A total of 1354 individuals (682 cases and 672 healthy controls) from Northwest Indian population were evaluated in the study. The epidemiological and clinical parameters were described in Table 1. The population enrolled in this study was genotyped for 12 Variants from 7 telomere maintenance genes including *OBFC1* (rs9419958), *TERF2* (rs4783704), *CSNK2A2* (rs74019828), *TEP1* (rs3093872, rs4982038, rs3093921), *TERT* (rs2736100), *TERC* (rs16847897, rs10936599, rs10936601) and *TERF1* (rs2010441, rs6982126) described as in Supplementary Table S1. After performing the quality control (QC) analysis^21,22^, the final data set remains with the 6 variants that pass the QC analyses and lie in HWE. Those variants were selected whose call rate was >90%. Randomly selected samples were also re-genotyped to check the precision of genotyping and showed no discrepancy. The variants which deviated from the HWE were also removed from the analyses part. The samples then further tested for association with T2D. Among the 6 variants, 5 variants were found to be significantly associated with T2D (Table 2). The associated variants were not found to be correlated with the age (Supplementary Table S2). The clinical characteristics of genotypes for each variant were compared and no variant has obvious effect on the clinical characteristics associated with T2D. The risk allele (C) of rs4783704 showed a *p*-value of 0.03 with low BMI that did not hold significance statistically after Bonferroni corrections (Supplementary Table S3). Yet it is interesting, as the number of males is more (n = 774) than females (n = 580) in the total population and literature has indicated that males develop T2D with lower BMI than females in early stages of life^23^. Therefore, it is an interesting perspective that needs further exploration in much larger sample size and in other Indian population groups. Genetic variations may influence the gene regulations by modifying the epigenome, transcription factor binding sites, and splicing factor binding sites^7,24,25^. The possible functional role of the variants in tissues that are chiefly involved in sugar metabolism that is, pancreas, pancreatic islets, adipose-subcutaneous tissue and muscle cells using databases GTEx v.7, UCSC, Haploreg v4.1, HSF (v.3.1) and ESE v.3^26–28^ was investigated. Result for each of the associated variant has been described separately below and summarized in Tables 2 and 3.

**Table 1:** Distribution of epidemiological parameters between Type 2 Diabetes patients and Healthy Controls populations from North India.

S.No.	Characteristic	Cases*	Controls	*p*-value
1.	Age	58.1 ± 8.02	58.58 ± 10.87	0.35
2.	Gender - Male	444 (65.1%)	330 (49.1%)	—
	Gender - Female	238 (34.9%)	342 (50.9%)	—
3.	BMI-kg/m^2^	26.4 ± 4.39	25.45 ± 4.01	9E-04
4.	Systolic Blood Pressure (SBP)-mmHg	135.07 ± 17.15	127.88 ± 14.32	7.22E-11
5.	Diastolic Blood Pressure (DBP)-mmHg	86.64 ± 10.45	82.68 ± 7.52	1.86E-10
6.	Blood Glucose (Post Prandial)-mg/dl	216.56 ± 68.66	120.32 ± 20.83	1.2E-107
7.	Triglycerides	256.98 ± 173.99	178.72 ± 109.2	4.84E-15
8.	High Density Lipoproteins (HDL)	49.92 ± 28.04	48.12 ± 21.9	0.3
9.	Very Low Density Lipoprotein (VLDL)	51.39 ± 34.79	35.98 ± 22.18	1.73E-14
10.	Cholesterol	170.89 ± 50.44	163.68 ± 47.92	0.04

*Other clinical conditions removed.

**Table 2:** Allelic Distribution. Logistic regression analysis of significant variants of genes in our study, adjusted for age, gender and BMI.

VARIANT	rs9419958		rs4783704		rs16847897		rs10936599		rs74019828	
NEAREST GENE (VARIANT)	*OBFC1*		*TERF2*		*TERC*		*TERC*		*CSNK2A2*	
POLYMORPHISM (ref/alt-GRCh38/hg38)	**T/C**		**C/T**		**G/C**		**C/T**		**G/A**	
ANCESTRAL ALLELE	**T**		**C**		**C**		**C**		**G**	
ALLELE DISTRIBUTION	**T**		**C**	**C**	**T**	**G**	**T**	**C**	**A**	**G**
CASES (n = 682)	0.04	0.96	0.13	0.87	0.43	0.57	0.16	0.84	0.07	0.93
CONTROLS (n = 672)	0.07	0.93	0.16	0.84	0.38	0.62	0.27	0.73	0.15	0.85
ALLELIC ODDS RATIO (95% CI)	1.98 (1.38–2.85)		1.32 (1.06–1.65)		1.23 (1.05–1.44)		1.76 (1.46–2.12)		2.10 (1.63–2.72)	
RISK ALLELE	**C**		**C**		**C**		**C**		**G**	
GENOTYPIC MODEL	**Recessive (CC vs TT** + **CT)**		**Recessive CC vs TT** + **CT)**		**Dominant (CC** + **GC vs GG)**		**Recessive (CC vs TT** + **CT)**		**Recessive (GG vs AA** + **GA)**	
*p*-VALUE*	5.8E-5		0.001		2.4E-9		1.63E-12		6.57E-9	
ODDS RATIO (95% CI)	2.30 (1.53–3.46)		1.52 (1.17–1.97)		2.23 (1.71–2.90)		2.44 (1.90–3.12)		2.37 (1.77–3.18)	

*Adjusted for Age, Gender, and BMI.

**Table 3:** Putative Role of the associated variants with T2D in studied population (North-west India) utilizing the information from the various databases including GTEX, UCSC genome browser and HSF.

Variant	Location of the variant w.r.t nearest gene	Allele Ref/Alt (Risk allele)*	eQTL gene	eQTL Tissue	eQTL sample size	eQTL NES	eQTL *p*-value	eQTL m-value	Putative role (cis- eQTL) of variant	Regulatory role of variant (ENCODE and Haploreg data)	Splicing effect^28,46^
rs9419958	1^st^ intron variant of *OBFC1/STN1*	T/C (C)	*OBFC1/STN1*	Pancreas	220	0.221	1.9E-3	1	Significant and Up regulation	H3K27ac_Enh/H3K4me3_Pro / H3K4me1_Enh/ H3K9ac_Pro/7_Enh/4_PromD2/TSSA/9_Txresg	Creation of ESE intronic site/ SF2/ASF (IgM-BRCA1)-91.23
				Adipose-subcutaneous	385	0.226	1.1E-4	1	Significant and Up regulation	H3K27ac_Enh/H3K4me3_Pro / H3K4me1_Enh/ H3K9ac_Pro/TSSAFlnk/3_PromD1	
				Skeletal Muscle	491	0.0057	0.9	0	Non-Significant	H3K27ac_Enh/H3K4me3_Pro / H3K4me1_Enh/ H3K9ac_Pro/TSSAFlnk/3_PromD1	
rs4783704	intergenic variant of *TERF2* and *CYB5B*	C/T (C)	N/A	Pancreas	N/A	N/A	N/A	N/A	N/A	22_PromP	Creation of ESE intronic site/ Creation of new site for SRp55–86.86 and 9G8–60.67
				Adipose-subcutaneous						H3K27ac_Enh/ H3K4me1_Enh/14_EnhA2	
				Skeletal Muscle						H3K27ac_Enh/ H3K9ac_Pro/16_EnhW1	
										Transcription factors binding sites including *TCF7L2, TCF12*; DNase hypersensitive #	
rs16847897	upstream to *TERC*; 7^th^ intron variant of *LRRC31*	G/C (C)	*LRRC34*	Pancreas	220	−0.169	0.1	0.924	Non-Significant	No Role w.r.t studied tissues	Creation of binding site for SF2/ASF (IgM-BRCA1)-75.85 and SRp55 site broken-100
				Adipose-subcutaneous	385	−0.208	3.7E-5	1	Significant and Down regulation		
				Skeletal Muscle	491	−0.139	0.03	0.965	Significant and Down regulation		
rs10936599	upstream to *TERC*; exon variant of *MYNN*	C/T (C)	*LRRC34*	Pancreas	220	−0.02	0.9	0.298	Non-Significant	H3K27ac_Enh/H3K4me3_Pro/ H3K4me1_Enh/H3K9ac_Pro (Pancreas) H3K27ac_Enh/H3K4me3_Pro/ H3K4me1_Enh/H3K9ac_Pro (Adipose-subcutaneous) H3K27ac_Enh/H3K4me3_Pro/ H3K4me1_Enh/H3K9ac_Pro (Skeletal Muscle)	Creation of ESE intronic site/Creation of new site for SRp40–82.10 and SC35–82.42
				Adipose-subcutaneous	385	−0.28	7.6E-8	1	Significant and Down regulation		
				Skeletal Muscle	491	−0.123	0.07	0.841	Non-Significant		
			*MYNN*	Pancreas	220	0.171	0.05	0.938	Non-Significant		
				Adipose-subcutaneous	385	0.099	0.03	0.97	Significant and Up regulation		
				Skeletal Muscle	491	0.106	2.2E-3	0.99	Significant and Up regulation		
rs74019828	4^th^ intron variant of *CSNK2A2*	G/A (G)	N/A	N/A	N/A	N/A	N/A	N/A	N/A	No Role w.r.t studied tissues	Creation of new binding site for SC35–75.17

*Risk allele in this study; NES – Normalized Effect Size in eQTL; m-value – posterior probability that effect exists in each tissue, ranges between 0 and 1; H3K27Ac_Enh – chemical modification (acetylation) of the histone proteins (H3) at lysine 27 and associated with transcriptional initiation and open chromatin structure (active enhancer); H3K4me3 – chemical modification (methylation) of the histone proteins (H3) at lysine 4, marks promoters that are active or poised to be activated; H3K4me1 – chemical modification (methylation) of the histone proteins (H3) at lysine 4 and is associated with enhancers, and downstream of transcription starts.; H3K9ac – chemical modification (acetylation) of the histone proteins (H3) at lysine 9 and is associated with transcriptional initiation and open chromatin structure; Enh – Enhancers; Pro – Promoters; TSSA – active transcription start site; TxReg – transcription regulatory; PromD1 – promoter downstream TSS; TSSAFlk – Flanking TSS; 22PromP – poised promoter; EnhW1 – weak enhancer; EnhA2 – active enhancer 2; the H3K4me1/2/3 and H3K36me2/3 are linked with genomic region which actively transcribing and H3K9me3, H3K27me3 and H4K20me3 with non-transcribing region; ESE – Exonic Splicing Enhancers; SR – Serine-Arginine rich proteins; 9G8, SC35 – SR splicing factor; SF2/ASF (IgM-BRCA1) – Serine-Arginine rich proteins; ^#^Supplementary Fig. S1.

### rs9419958

The variant rs9419958 is located in the intronic region of the *OBFC1* gene, a component of CST complex which assists in the efficient replication of telomeres and negatively regulates the telomere length by inhibiting the action of telomerase^29,30^. The variant was reported to be associated with telomere attrition^31^. In our study, the major allele (C) of variant rs9419958 (T/C) showed significantly increased risk for T2D with odds ratio (OR) of 2.3 (95% CI = 1.53–3.46) and *p-*value of 6.5E-5 (Table 2). As a result of cis-eQTL analysis, the risk allele (C) is associated with up regulation of the expression of the gene in pancreas (*p-*value = 1.9E-3 and normalized effect size (NES) = 0.221) and adipose-subcutaneous tissue (*p-*value = 1.1E-4 and NES = 0.226). The gene is responsible for terminating the telomerase activity at the telomeric ends^32^ hence it is possible that the overexpression of the gene might lead to early termination of telomerase activity leading to attrition^30,33^. The locus showed the presence for histone marks (H3K27ac_Enh/H3K4me3_Pro/H3K4me1_Enh/H3K9ac_Pro), promoter and transcription regulatory activity, active transcription start site (TSS) (from chromatin 15 and 25 state model) and change in splicing factor binding sites of exonic splicing enhancers (ESE) intronic site and SF2/ASF (IgM-BRCA1) suggesting the locus plays an important role in epigenetic regulation (Table 3 and Supplementary Fig. S2).

### rs4783704

The variant rs4783704 is an intergenic variant (*TERF2* and *CYB5B*), upstream to *TERF2* gene. *TERF2* is a component of shelterin complex and implicated in the formation and stability of T-loop^15^. The variant was reported to be associated with T2D in a candidate gene association study conducted in Caucasian women^16^. In the present study, the major allele (C) of variant rs4783704 (C/T) showed significant risk for T2D with odds ratio (OR) of 1.52 (95% CI = 1.17–1.97) and *p-*value of 0.001 (Table 2). The region showed the presence of histone marks (H3K27ac_Enh/H3K4me1_Enh/H3K9ac_Pro), promoter activity (22_PromP), enhancer activity (14_EnhA2), DNase hypersensitivity, binding sites for splicing factors, SRp55 and 9 G and a strong binding site for transcription factors (Table 3 and Supplementary Figs. S1 and S2). One of the transcription factors is *TCF7L2* (Supplementary Fig. S1) which is worldwide robustly associated with T2D and involved in several pathways including blood glucose homeostasis, insulin secretion and biosynthesis pathway and wingless type (wnt) signalling^34,35^.

### rs16847897

The variant rs16847897 is located upstream to Telomerase RNA component (*TERC*) and in the intronic region of the Leucine-rich repeat-containing protein 31 (*LRRC31*). *TERC* is a component of telomerase complex and act as RNA template during the replication process of telomeres^36^. *LRRC31* belongs to a superfamily of *LRRC – LRCC31, LRCC34* and *LRRIQ4*. The superfamily is involved in diverse roles including cell cycle regulation, chromosome stability, apoptosis and DNA repair^37^. Our study showed that the major allele (C) of variant rs16847897 (G/C) is associated with significant increased risk for T2D with odds ratio (OR) of 2.23 (95% CI = 1.71–2.9) and *p-*value of 2.4E-9 (Table 2). A study showed that the risk allele (C) is a part of the *TERC* haplotype GTC (rs12696304-G, rs10936601-T, and rs16847897-C) which is associated with increased risk of T2D and telomere attrition^15,38,39^. Our *in silico* analysis revealed that the risk allele (C) of variant rs16847897 is down regulating the expression of the *LRRC34* gene in adipose-subcutaneous tissue (*p-*value = 3.7E-5 and NES = −0.208) and skeletal muscle (*p-*value = 0.03NES = −0.139) and also is a binding site for splicing factors (SF2/ASF (IgM-BRCA1) and SRp55) (Table 3 and Supplementary Fig. S2).

### rs10936599

The variant rs10936599 is located upstream to *TERC* and in the 2^nd^ exon of Myoneurin (*MYNN*) gene. *MYNN* belongs to BTB/POZ and zinc finger domain-containing protein family that is involved in the control of developmental events, and activation and suppression of transcription of distinct genes^40^. The variant has been found to be associated with telomere length attrition^10^. So far, only one candidate gene association study with T2D has been conducted where this variant did not show association with T2D in Caucasian women^16^. In the present study, the major allele (C) of variant rs10936599 (C/T) is significantly associated with high risk for T2D with odds ratio (OR) of 2.44 (95% CI = 1.9–3.12) and *p-*value of 1.63E-12 (Table 2). As a result of our *in silico* analysis, resistant allele (T) of this variant significantly down regulates the expression of *LRRC34* gene in adipose-subcutaneous tissue (*p-*value = 7.6E-8 and NES = −0.28). It is also shown to up regulate the expression of *MYNN* gene in adipose-subcutaneous tissue (*p-*value = 0.03 and NES = 0.099) and skeletal muscle (*p-*value = 2.2E-3 and NES = 0.106). The locus also observed to have histone marks (H3K27ac_Enh/H3K4me3_Pro/H3K4me1_Enh/H3K9ac_Pro) and splicing factor binding sites (ESE intronic site, SRp40 and SC35) (Table 3 and Supplementary Fig. S2).

### rs74019828

The variant rs74019828 is located at the 8^th^ intron of the Casein Kinase 2 Alpha 2 (*CSNK2A2*). *CSNK2A2* gene encodes a constitutively active catalytic subunit of a serine/threonine protein kinase enzyme. It plays a role in the regulation of telomere length by phosphorylating *TRF1*, which enhances its binding to telomeres and negatively regulates the telomere length^18^. The variant is associated with telomere attrition in a Punjabi diabetic Sikh cohort^18^. In our study, the major allele (G) of variant rs74019828 (G/A) was significantly associated with higher risk for T2D with odds ratio (OR) of 2.37 (95% CI = 1.77–3.18) and *p-*value of 6.57E-9 (Table 2). *In silico* analysis showed that the risk allele is associated with the formation of new splice sites for the splicing factor, SC35 (Table 3 and Supplementary Fig. S2).

The subgroup analysis by gender was performed. Here, it was observed that the association of variants varies among males and females in the studied population (Supplementary Table S4). The significantly associated variants with T2D in Northwest Indian population were also found to be significantly associated with T2D in males sub-group. However, in females only the two variants namely, rs10936599 and rs74019828 showed significant association with T2D that needs further exploration in larger sample size for conclusions.

The existence of interaction among the variants of telomere maintenance genes for the risk of developing T2D in the studied population was explored. The risk genotype of the associated variants was compared with the other possible combinations in cases and controls. The interactions were carried out stepwise. Interaction of variants, with respect to their location, i.e. within three complexes namely, telomerase, CST and shelterin was carried out followed by pairwise interaction of variants, irrespective of the location in the genes. The interaction among the risk genotypic combination in associated variants involved in the telomere maintenance genes was found to be significant. The variants (rs16847897 and rs10936599) of telomerase complex showed highly significant association (*p-*value = 9.4E-34, OR = 5.04 (3.88–6.55) at 95%CI) with T2D. A significant association (*p-*value = <0.001, OR = 6.03 (4.59–7.91) at 95%CI) was also found among the variants (rs16847897, rs10936599 and rs9419958) of telomerase and CST complexes. Likewise, the variants (rs16847897, rs10936599, rs9419958, rs74019828 and rs4783704) of telomerase, CST and shelterin complexes also showed significant association (*p-*value = 1E-36, OR = 8.63 (6.19–12.04) at 95%CI) with T2D. The risk for T2D ranges from 1.85 to 8.63 folds (Supplementary Table S5).

## Conclusion

Worldwide so far, 129 loci have been associated with T2D by candidate gene association studies and GWAS^7^, however, these genes are involved predominantly in insulin secretion/action pathway. The high incidence of T2D necessitates the exploration of other pathways which could lead to T2D predisposition and Telomere maintenance pathway is one of these^15^. In present study, the association of the variants of telomere maintenance gene with T2D was explored and five variants showed significant association with T2D (ranging OR = 1.52–2.44 and *p-*value ≤ E-3). The subgroup analysis by gender showed interesting results. All the variants showed significant association in the male subgroup however, only two variants namely, rs10936599 and rs74019828 remain significant in the female group. It is an interesting perspective and should be explored in future studies in larger sample size and independent population groups. *In silico* analyses of these variants indicated the presence of one or more regulatory sites (histone marks/promoter and enhancer activity/transcription factor binding sites/splicing factor binding sites) and three variants (rs9419958 of *OBFC1*, rs16847897 and rs10936599 of *TERC*) showed significant cis-eQTL effect. The interaction analysis also showed significant association with T2D (*p-*value ranging from E-06 to E-36) and 1.85 to 8.63 fold risk for developing T2D.

This first study from India (Northwest) elucidates the role of telomere maintenance genes in T2D. The findings from Northwest India suggest the importance of telomere maintenance pathway in T2D susceptibility and propose replication of it worldwide in all population group subsets for better understanding of the underlying mechanism played by the telomere maintenance genes in T2D etiology. Moreover, the putative functional annotations of these variants indicate these might have strong regulatory role in telomere maintenance pathway which further warrants functional validation studies of these variant.

## Materials and Methods

### Ethical statement

The present study was conducted after attaining approval by Institutional Ethics Review Board (IERB) of Shri Mata Vaishno Devi University (SMVDU). The study was performed in accordance with the relevant guidelines and regulations. A written informed consent was acquired from all the participants enrolled in the present study.

### Subjects

A total of 1354 randomly collected samples were analysed in the present study which includes 682 diabetic individuals and 672 healthy individuals belonging to Northwest Indian population groups. A diagnostic criterion was made according to the International Diabetes Federation (IDF). Epidemiological characteristics were summarized in Table 1. The control group has postprandial glucose levels below 11 mmol/ L.

### Selection of variants and genotyping

In the present study, the variants were selected (Supplementary Table S1) which have been implicated in telomere length attrition via genome-wide association studies (GWAS) and replication studies using the candidate gene approach. All the experiment work was conducted as per the set guidelines and regulations by Institutional Ethics Review Board (IERB), Shri Mata Vaishno Devi University (SMVDU). With written informed consent from the subjects, 2 ml of blood by venepuncture was collected. Genomic DNA extraction was performed by a conventional phenol-chloroform method and FlexiGene® DNA kit, QIAGEN (catalogue No. 51206) method. The quantity and quality check of genomic DNA was analysed by carrying out UV spectrophotometer (eppendorf Biospectrometer®, Hamburg Germany) analysis and Gel electrophoresis respectively. The genomic DNA was stored at 4 °C till further use at a concentration of 10 ng/µl. Genotyping was carried out on a high-throughput Agena MassARRAY platform (The MassARRAY® System by Agena Bioscience™, San Diego, CA). A customized panel of 12 variants from critical telomere maintenance pathway genes was made. The panel was made by using Agena Bioscience Assay Design Suite (version 2.0). The sequence of primers has been provided in Supplementary Table S6. The recommended (standardized) protocol was followed for the genotyping. The genotype calls were analyzed by using the software Sequenom Typer 4.0. All genotype calls were cross checked to evaluate and exclude the call errors via spectrograms. The subjects were excluded from the study if the missing genotypes were higher than 10%. Those that deviated from the Hardy-Weinberg Equilibrium (HWE) (*p-*value <0.05) were also excluded from the study.

### Statistical analyses

Clinical characteristics between cases and controls were compared by t-test. Statistical analyses on the genotype data was performed by using the PLINK v. 1.07 and gPLINK^41^ and IBM SPSS statistics 20 software^42^. Each variant was tested for Hardy-Weinberg equilibrium using chi-square test. Correlation of variants with age was performed by Pearson Correlation analysis. The association of variants with T2D was confirmed by binary logistic regression adjusted for confounding factors like age, gender and Body Mass Index (BMI). The odds ratios (ORs) were calculated with respect to the risk allele found in this study. The comparison of clinical characteristics of different genotypes for each variant was performed by one way ANOVA, adjusted for age and gender. The interaction analysis was performed by logistic regression analysis using IBM SPSS statistics 20 software and adjusted for age, gender and BMI.

### Putative functional role of the variants

The combined expression Quantitative Trait Loci (eQTL) analysis of the variants was explored by using University of California Santa Cruz (UCSC) Genome Browser (https://genome.ucsc.edu) and GTEx portal (https://www.gtexportal.org) ^43,44^. The transcriptional regulatory role like histone modifications, DNase hypersentivity and binding sites for the transcription factor was analysed by UCSC Genome Browser, Encyclopedia of DNA Elements (ENCODE) (V3) and Haploreg v4.1 database^26,45^. The effect of variant on splicing by using the web tool Human Spicing Finder (HSF) 3.1and ESE finder (3.0)^28,46^ was also analysed.

## Supplementary information

Telomere Maintenance Genes are associated with Type 2 Diabetes Susceptibility in Northwest Indian Population Group.
