# Supplementary material for: Telomere Maintenance Genes are associated with Type 2 Diabetes Susceptibility in Northwest Indian Population Group
Source: Sci Rep. 2020 Apr 15;10:6444. doi: 10.1038/s41598-020-63510-w (PMC7160122; doi:10.1038/s41598-020-63510-w)
Supplement: Supplementary file 1 — Telomere Maintenance Genes are associated with Type 2 Diabetes Susceptibility in Northwest Indian Population Group. [file 41598_2020_63510_MOESM1_ESM.pdf]

Telomere Maintenance Genes are associated with Type 2 Diabetes Susceptibility in Northwest Indian Population Group

Itty Sethi[a], Varun Sharma[a], Indu Sharma[a], Gurvinder Singh[b], Gh. Rasool Bhat[a], A.J.S Bhanwer[b], Swarkar Sharma[a], and Ekta Rai[a]

Affiliations:

[a]Human Genetics Research Group, School of Biotechnology, Shri Mata Vaishno Devi University Katra, J&K, 182320.

[b]Department of Human Genetics, Guru Nanak Dev University, Amritsar, 143005, Punjab, India.

Corresponding Authors:

Dr. Swarkar Sharma, Ph.D.

Human Genetics Research Group,

School of Biotechnology

Shri Mata Vaishno Devi University,

Katra, Jammu and Kashmir, 182320

Tel: 01991-285535 // 285524 // 285634 // 285699 extn: 2533

Email: swarkar.sharma@smvdu.ac.in

Dr. Ekta Rai

Human Genetics Research Group,

School of Biotechnology

Shri Mata Vaishno Devi University,

Katra, Jammu and Kashmir, 182320

Tel: 01991-285535 // 285524 // 285634 // 285699 extn: 2533

Email: ekta.rai@smvdu.ac.in

SUPPLEMENTARY INFORMATION

Supplementary Figures

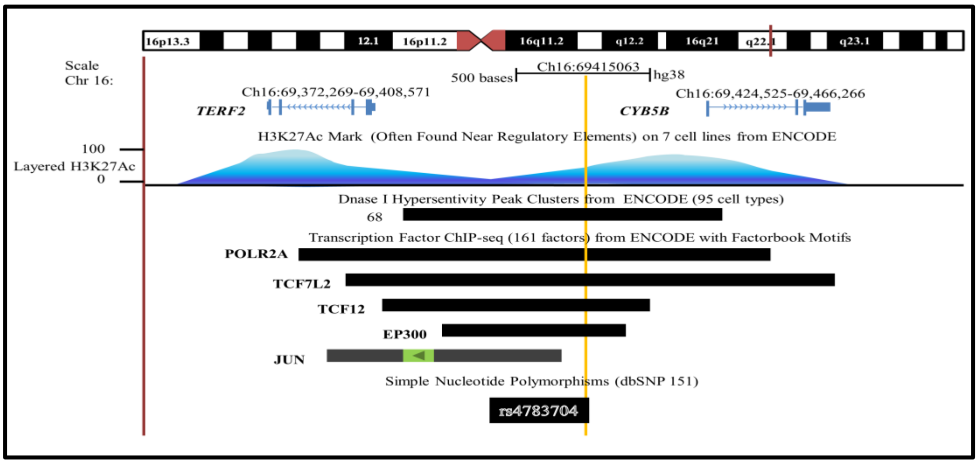

Supplementary Figure S1: Representation of the variant rs4783704 from UCSC Genome browser. It is an intergenic variant of *TERF2* and *CYB5B* and is a binding site for various transcription factors including POLR2A (Cluster score-983), TCF7L2 (Cluster score-1000), TCF12 (Cluster score-1000), EP300 (Cluster score-1000), and JUN (Cluster score-631). The region is DNase Hypersensitive and showed the presence of histone marks. The black boxes of transcription factor corresponds to the maximum signal strength observed for transcription factor

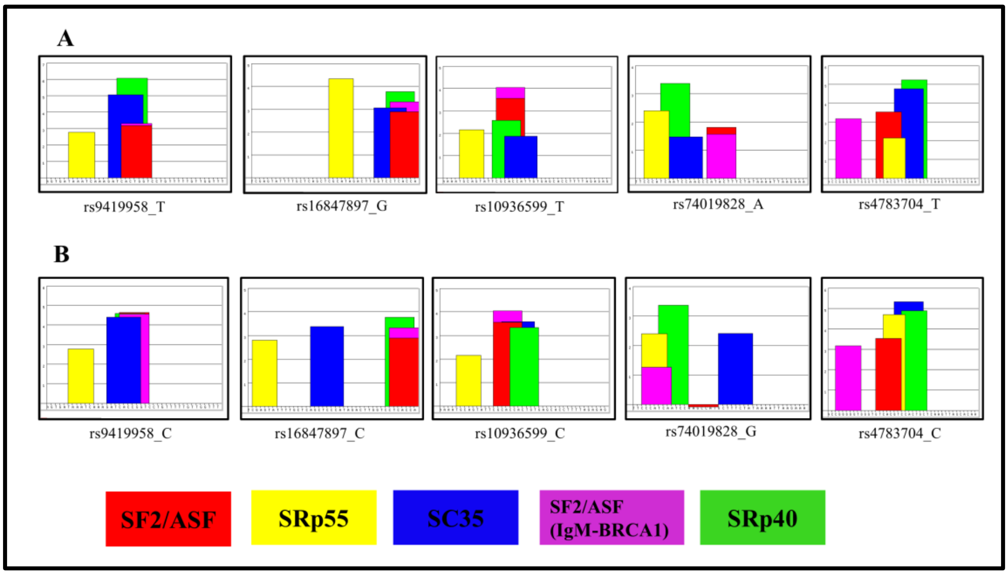

Supplementary Figure S2: Effect of polymorphism on the Exonic Splicing Enhancers (ESEs) according to ESE prediction tool. ESE finder enables to identify the potential ESE sites. The height of the colored bars represents the motif scores and the width of the bars indicates the length of the motif. Bars in red, yellow, blue, purple and green indicate potential binding sites for Serine-Arginine (SR) proteins SF2/ASF, SRp55, SC35, SF2/ASF (IgM-BRCA1) and SRp40, respectively. Panel A represents the ESE sequence with the allele not posing risk in the studied population and panel B represents the ESE sequence with the risk allele in the studied population. From the figure, we can predict that there is a change in the

potential ESE sites as can be seen from change in the bars (change in the potential splicing sites) that could lead to the disease susceptibility.

Supplementary Tables

Supplementary Table S1: Details of the variants selected for the study

S.No.	SNP	Chromosome: position	Location of the Variant w.r.t Gene	Candidate Gene and its Functional role
1.	rs16847897	3:169850328	upstream to *TERC*; intron variant of *LRRC31*	*TERC*: Encodes for non-coding RNA that provides template for telomere replication
2.	rs10936599	3:169774313	upstream to *TERC*; exon variant of *MYNN*	
3.	rs10936601	3:169810661	upstream to *TERC*; intron variant of *LRRC34*	
4.	rs2736100	8:1286401	intron variant of *TERT*	*TERT*: Encodes for an enzyme called telomere reverse transcriptase, helps in telomere replication
5.	rs74019828	16:58175370	intron variant of *CSNK2A2*	*CSNK2A2*: Phosphorylates *TERF1* protein and promotes its binding with the telomeric DNA thereby negatively regulating the telomere length by inhibiting telomerase action
6.	rs4783704	16:69410563	intergenic variant of *TERF2* and *CCYBB*	*TERF2*: Component of Shelterin complex, negatively regulates the telomere length by inhibiting the telomerase binding to the telomeric DNA
7.	rs2010441	8: 73005929	upstream variant of *TERF1*	*TERF1*: Component of shelterin complex, inhibits the telomerase
8.	rs6982126	8:73027388	intron variant of *TERF1*	
9.	rs3093872	14:20343173	non-coding transcript variant of *RPPH1* and downstream to *TEP1*	*TEP1*: Component of telomerase, aids in the stability of the telomerase complex
10.	rs4982038	14:20394383	intron variant of *TEP1*	
11.	rs3093921	14:20354149	missense variant of *PARP2*; downstream to *TEP1*	
12.	rs9419958	10:103916188	intron variant of *OBFC1/STN1*	*OBFC1*: Component of CST complex, aids in telomere replication and negatively regulates the telomerase action

Variants of telomere maintenance genes were studied in North-West population of India with total sample size, n=1354 (cases = 682 and controls = 672)

Supplementary Table S2: Correlation analysis of associated variants involved in the telomere maintenance gene complex in the studied population with age

Variant	rs9419958			rs4783704			rs16847897			rs10936599			rs74019828		
Nearest gene	*OBFC1*			*TERF2*			*TERC*			*TERC*			*CSNK2A2*		
Genotype	TT	TC	CC	TT	TC	CC	GG	GC	CC	TT	TC	CC	AA	AG	GG
Pearson Correlation	0.05			0.04			0.004			-0.04			0.001		
P-value	0.1			0.1			0.9			0.2			0.9		

Supplementary Table S3: One way ANOVA of significant Variants with quantitative traits

Variant	rs9419958			rs4783704			rs16847897			rs10936599			rs74019828		
Nearest gene	*OBFC1*			*TERF2*			*TERC*			*TERC*			*CSNK2A2*		
	MEAN±SE			MEAN±SE			MEAN±SE			MEAN±SE			MEAN±SE		
Genotype	TT	TC	CC	TT	TC	CC	GG	GC	CC	TT	TC	CC	AA	AG	GG
BMI	27.9 ±1.7	27.4 ±0.8	26.2 ±0.3	28.5 ±2.3	25.4 ±0.4	26.7 ±0.3	26.4 ±0.4	26.2 ±0.4	27.1 ±0.6	25.8 ±0.6	26.7 ±0.4	26.3 ±0.4	29.8 ±0.9	26.7 ±0.4	26.1 ±0.3
P-value	0.3			0.03			0.47			0.5			0.08		
Age of onset	43.6 ±2.5	47.9 ±1.4	47.9 ±0.5	51 ±2.9	47.5 ±1.0	47.9 ±0.6	48.02 ±0.7	46.99 ±0.7	50.3 ±1.6	49.3 ±1.5	47.9 ±0.9	47.5 ±0.6	48.5 ±3.9	47.5 ±0.9	48.0 ±0.6
P-value	0.5			0.5			0.09			0.5			0.9		
Blood glucose (random) mg/dl	248 ±40.6	224.6 ±13.8	213.8 ±4.8	228.8 ±22	215.2 ±8.9	215.7 ±5.3	218.2 ±7.0	214.8 ±6.7	214.4 ±12.8	212.7 ±12.4	216.9 ±7.4	217.2 ±6.4	204.7±21.6	209.8 ±6.9	219.5 ±5.8
P-value	0.5			0.9			0.9			0.9			0.6		
SBP (mmhg)	128.8 ±8.3	132.5 ±3.1	135.3 ±1.1	139.9 ±5.9	133.9 ±1.7	134.6 ±1.2	134.7 ±1.6	135.1 ±1.5	134.1 ±2.4	130.6 ±2.6	135.3 ±1.8	135.3 ±1.3	130 ±7.4	134.2 ±1.9	135 ±1.2
P-value	0.5			0.6			0.9			0.3			0.7		
DBP (mmhg)	82.5 ±6.3	83 ±1.7	87.1 ±0.7	88.5 ±4.4	85.8 ±1.0	86.6 ±0.8	86.8 ±1.1	86.5 ± 0.8	85.8 ±1.6	84.2 ±1.7	87.3 ±1.0	86.5 ±0.9	87 ±6.2	87.6 ±1.3	85.9 ±0.7
P-value	0.1			0.7			0.9			0.3			0.5		

Adjusted for age and gender

Supplementary Table S4: Subgroup analysis by gender of the variants of Telomere Maintenance Genes

VARIANT	rs9419958		rs4783704		rs16847897		rs10936599		rs74019828	
NEAREST GENE (VARIANT)	*OBFC1*		*TERF2*		*TERC*		*TERC*		*CSNK2A2*	
POLYMORPHISM (ref/alt-GRCh38/hg38)	T/C		C/T		G/C		C/T		G/A	
ALLELE DISTRIBUTION	T	C	T	C	C	G	T	C	A	G
CASES										
MALES (n=444)	0.02	0.98	0.12	0.88	0.45	0.55	0.14	0.86	0.07	0.93
FEMALES (n=238)	0.06	0.94	0.14	0.86	0.40	0.60	0.20	0.80	0.09	0.91
CONTROLS										
MALES (n=330)	0.06	0.94	0.16	0.84	0.38	0.62	0.24	0.76	0.14	0.86
FEMALES (n=342)	0.07	0.93	0.16	0.84	0.38	0.62	0.29	0.71	0.15	0.85
RISK ALLELE	C		C		C		C		G	
GENOTYPIC MODEL	Recessive (CC vs TT+CT)		Recessive (CC vs TT+CT)		Dominant (CC+GC vs GG)		Recessive (CC vs TT+CT)		Recessive (GG vs AA+GA)	
p-VALUE*/ ODDS RATIO (95% CI)										
MALES	1.7E-04/ 2.98 (1.69-5.27)		0.01/ 1.54 (1.10-2.15)		9.29E-09/ 2.76 (1.95-3.89)		7.62E-09/ 2.58 (1.87-3.56)		2.5E-06/2.49 (1.71-3.66)	
FEMALES	-		-		-		6.5E-04/ 1.64 (1.23-2.18)		0.003/ 1.8 (1.22-2.66)	

*Adjusted for age, gender and BMI

Supplementary Table S5: Interaction analysis among the variants of telomere maintenance genes

S.No.	Genotypic combination	Status	Cases (n=632)	Controls (n=651)	p-value	odds ratio (95% CI)
1.	rs16847897 + rs10936599	Risk	0.61	0.24	9.4E-34	5.04 (3.88-6.55)
	All other	Mixed	0.39	0.76		
2.	rs16847897 + rs10936599 + rs9419958	Risk	0.60	0.21	1 E-36	6.03 (4.59-7.91)
	All other	Mixed	0.40	0.79		
3.	rs16847897 + rs10936599 + rs9419958 + rs74019828	Risk	0.58	0.16	1 E-36	8.28 (6.16-11.15)
	All other	Mixed	0.42	0.84		
4.	rs16847897 + rs10936599 + rs74019828 + rs4783704 + rs9419958	Risk	0.48	0.11	1E-36	8.63 (6.19-12.04)
	All other	Mixed	0.52	0.89		
5.	rs16847897 + rs9419958	Risk	0.78	0.57	2.41E-13	2.61 (2.02-3.37)
	All other	Mixed	0.22	0.43		
6.	rs16847897 + rs74019828	Risk	0.73	0.47	7.1E-20	3.19 (2.49-4.10)
	All other	Mixed	0.27	0.53		
7.	rs16847897 + rs4783704	Risk	0.64	0.43	7.15E-13	2.39 (1.89-3.04)
	All other	Mixed	0.36	0.57		
8.	rs10936599 + rs9419958	Risk	0.74	0.47	8.46E-20	3.18 (2.48-4.08)
	All other	Mixed	0.26	0.53		
9.	rs10936599 + rs74019828	Risk	0.68	0.40	4.28E-22	3.37 (2.63-4.31)
	All other	Mixed	0.32	0.60		
10.	rs10936599 + rs4783704	Risk	0.61	0.39	8.89E-15	2.58 (2.03-3.27)
	All other	Mixed	0.39	0.61		
11.	rs9419958 + rs74019828	Risk	0.81	0.63	8.07E-13	2.67 (2.04-3.49)
	All other	Mixed	0.19	0.37		
12.	rs9419958 + rs4783704	Risk	0.74	0.62	1.4E-06	1.85 (1.44-2.38)
	All other	Mixed	0.26	0.38		
13.	rs74019828 + rs4783704	Risk	0.68	0.52	6.04E-09	2.04 (1.61-2.59)
	All other	Mixed	0.32	0.48		

1 - Telomerase complex variant interaction; 2 - telomerase and CST complex variant interaction; 3 - telomerase, CST and CSNK2A2 variant interaction; 4 - telomerase, shelterin and CST complex variant interaction; **adjusted for age, gender and BMI

Supplementary Table S6: List of Primers and UEPs Sequence of the Selected Variants in the Study for MassARRAY Genotyping

S.No	Variant	Gene	Forward Primer Sequence	Reverse Primer Sequence	Amplicon Length	Universal Extended Primer Sequence
1.	rs16847897	*LRRC31/ TERC*	ACGTTGGATGTTGCCC TCCTTTTCGGCCT	ACGTTGGATGAGGAC ATGGTGAGGACCAAG	120	GTGAGGACCAAGTCTATG
2.	rs10936599	*MYNN/ TERC*	ACGTTGGATGCAAGG GTAAAATTCCATTCTG	ACGTTGGATGCGCTG TTTGTTCAGTCTCTC	102	ggaAGTCTCTCTAAAAGGT GCTCACA
3.	rs10936601	*LRRC34/ TERC*	ACGTTGGATGAGGAT GATCAAAGGTGTTCC	ACGTTGGATGTGAGA AGTGTTGTGTCTGTG	120	AATAGGTCTTATTATCAA GTAAGT
4.	rs2736100	*TERT*	ACGTTGGATGACAAA GGAGGAAAAGCAGGG	ACGTTGGATGTGACA CCCCCACAAGCTAAG	110	ggggaTTTTCCGTGTTGAGT GTTTCT
5.	rs74019828	*CSNK2A2*	ACGTTGGATGATCTGT TTTCCCATCAATCC	ACGTTGGATGGCTTG AGTCTCTCTTTCAAT	119	CCATCAATCCAAGCCAT
6.	rs4783704	*TERF2/ CYB5B*	ACGTTGGATGACCTA ACCTGAACTTAACCG	ACGTTGGATGCTCCA AACCTCGATGCTTTC	116	agggGTGCTAACTTGAGCA GTG
7.	rs2010441	*TERF1*	ACGTTGGATGTCTAG GCAGTGAGCAAGGTG	ACGTTGGATGGAGTG ATGCTAACAGTAACC	100	AGGAGTTTGGTGGGG
8.	rs6982126	*TERF1*	ACGTTGGATGCTACA TGTTCTCATTATGTG	ACGTTGGATGCAGTG AGCTTATGCTTAGAG	116	gATCTTAAAATACTAGAC TCTTAGTACA
9.	rs3093872	*RPPH1/ TEP1*	ACGTTGGATGTTCGCT GGCCGTGAGTCTGT	ACGTTGGATGTCAGA CTGGGCAGGAGATG	118	gGCCTCCTTTGCCGGA
10.	rs4982038	*TEP1*	ACGTTGGATGGACTG CCTTCAAAACCTAGC	ACGTTGGATGTGACC AATGTAGCCACTAGC	114	gtatCAGTTGAAATATGCTA GTGA
11.	rs3093921	*PARP2/ TEP1*	ACGTTGGATGAATCT CCCTTGAAGCCAGAG	ACGTTGGATGTTCCA TGGCCTGAACATTAC	103	aCCAGAGTCACAGCTAG
12.	rs9419958	*OBFC1/ STN1*	ACGTTGGATGCTTGG AAACATTCTCACCAG	ACGTTGGATGTCTAC CTGTAGGCAAAAGAC	118	cccACCAACAAAAACAGG ACC
